# Supplementary material for: Structural insights into the molecular mechanisms of myasthenia gravis and their therapeutic implications
Source: eLife. 2017 Apr 25;6:e23043. doi: 10.7554/eLife.23043 (PMC5404922; doi:10.7554/eLife.23043)
Supplement: Supplementary file 1. — DOI: http://dx.doi.org/10.7554/eLife.23043.015 [file elife-23043-supp1.docx]

|  | Fab35/Human nAChR α1 ECD/α-Btx  (PDB ID, 5HBT) | Fab35/Mouse nAChR α1 ECD/α-Btx  (PDB ID, 5HBV) |
| --- | --- | --- |
| Data collection |  |  |
| **Space Group** | C2 | C2 |
| **Cell dimensions** |  |  |
| ***a, b, c* (Å)** | 160.01 42.14 136.49 | 159.91 42.02 137.58 |
| ***β* (°)** | 117.05 | 116.46 |
| **Resolution (Å)** | 50.00­­–2.60 (2.64–2.60)^a^ | 50.00–2.70 (2.75–2.70) |
| ***R*_sym_ (%)** | 12.3 (100.9) | 12.5 (86.4) |
| ***R*_meas_ (%)** | 14.3 (118.0) | 14.7 (102.8) |
| ***I*/σ*I*** | 15.2 (1.5) | 13.3 (1.3) |
| **Completeness (%)** | 100 (100.0) | 99.5 (96.1) |
| **Multiplicity** | 3.7 (3.7) | 3.6 (3.3) |
| **No. unique Reflections** | 25,189 | 23,334 |
| **CC_1/2_ in the highest shell** | 0.568 | 0.537 |
|  |  |  |
| Refinement |  |  |
| **Resolution (Å)** | 50.01–2.61 | 50.01–2.70 |
| **No. reflections** | 23,958 | 21,877 |
| ***R*_work_/*R*_free_ (%)** | 20.7/26.0 | 22.7/26.8 |
| **No. atoms** |  |  |
| **nAChR α1 ECD** | 1,836 | 1,843 |
| **α-Btx** | 547 | 547 |
| **Heavy chain** | 1,671 | 1,646 |
| **Light chain** | 1,628 | 1,623 |
| **Water** | 102 | 46 |
| **B-factors (Å^2^)** |  |  |
| **nAChR α1 ECD** | 48.58 | 52.64 |
| **α-Btx** | 84.01 | 62.37 |
| **Heavy chain** | 62.49 | 93.11 |
| **Light chain** | 56.90 | 70.62 |
| **Water** | 44.42 | 42.56 |
| **R.m.s deviations** |  |  |
| **Bond length (Å)** | 0.008 | 0.007 |
| **Bond angles (°)** | 1.341 | 1.216 |
| **Ramachandran plot (%)^b^** |  |  |
| **Favored region** | 95.8 | 95.5 |
| **Allowed region** | 4.2 | 4.5 |
| **Outlier region** | 0.0 | 0.0 |

**Supplementary file 1. The statistics of data collection and structure refinement for human and mouse Fab35/nAChR α1 ECD/α-Btx ternary complexes**

^a^ Highest resolution shell shown in parentheses.

^b^ As defined in MolProbity
