# Supplementary material for: Structural insights into the molecular mechanisms of myasthenia gravis and their therapeutic implications
Source: eLife. 2017 Apr 25;6:e23043. doi: 10.7554/eLife.23043 (PMC5404922; doi:10.7554/eLife.23043)
Supplement: Supplementary file 2. — Residues at the Fab35/human nAChR α1 (α211) interface were mapped using the contact program in CCP4 with a 4.5 Å distance cutoff (Winn et al., 2011). For each residue of nAChR α1 involved in antibody binding (Chain B/α211 listed in the first column), its interacting residues from the light chain (Chain C listed in the second column) and the heavy chain (Chain D listed in the third column) of Fab35 are listed in the corresponding row. Residues on the N-terminal helix is highlighted in yellow and residues on the MIR loop is highlighted in light green. Note that several nAChR α1 residues, including R6, K10, N68 and D71/Y72 (red font), contact a large number of antibody residues. These residues can be considered as ‘hotspots’ of the binding interface. Y72 can be considered as part of the Asp71 ‘hotspot’ (see the text). DOI: http://dx.doi.org/10.7554/eLife.23043.016 [file elife-23043-supp2.docx]

| **α211 (B)** | **Light Chain (C)** | **Heavy Chain (D)** |
| --- | --- | --- |
| H3 |  | T57, V58 |
| R6 |  | W52, D54, G56, T57 |
| L7 |  | A103 |
| K10 |  | W52, D53, D54, R100, R102, A103 |
| L11 |  | A103 |
| D14 |  | R102 |
| Y15 |  | R102 |
| E23 | K50 |  |
| Y63 | Y32, K50 |  |
| N64 |  | R102 |
| K66 | Y32 | A103, I104 |
| W67 | I92 | A103, I104 |
| N68 | Y91, I92, N93, G94, Y95 |  |
| P69 | I92, N93 |  |
| D70 | G94, Y95 | W47, V58 |
| D71 | Y95 | W47, R50, W52, V58, A103, N105 |
| Y72 |  | W52, V58, A103, N105 |
| G73 |  | V58 |

**Supplementary file 2. Contacting residues at the Fab35/human nAChR α1 ECD interface**

Residues at the Fab35/human nAChR α1 (α211) interface were mapped using the contact program in CCP4 with a 4.5Å distance cutoff (1). For each residue of nAChR α1 involved in antibody binding (Chain B/α211 listed in the first column), its interacting residues from the light chain (Chain C listed in the second column) and the heavy chain (Chain D listed in the third column) of Fab35 are listed in the corresponding row. Residues on the N-terminal helix is highlighted in yellow and residues on the MIR loop is highlighted in light green. Note that several nAChR α1 residues, including R6, K10, N68 and D71/Y72 (red font), contact a large number of antibody residues. These residues can be considered as “hotspots” of the binding interface. Y72 can be considered as part of the Asp71 “hotspot” (see the text).

1. Winn MD, Ballard CC, Cowtan KD, Dodson EJ, Emsley P, Evans PR, Keegan RM, Krissinel EB, Leslie AG, McCoy A, McNicholas SJ, Murshudov GN, Pannu NS, Potterton EA, Powell HR, Read RJ, Vagin A, Wilson KS. Overview of the CCP4 suite and current developments. Acta Crystallogr D Biol Crystallogr. 2011;67(Pt 4):235-42.
